# Neural substrates of socioemotional self-awareness in neurodegenerative disease

**DOI:** 10.1002/brb3.211

**Published:** 2014-01-13

**Authors:** Marc Sollberger, Howard J Rosen, Tal Shany-Ur, Jerin Ullah, Christine M Stanley, Victor Laluz, Michael W Weiner, Stephen M Wilson, Bruce L Miller, Katherine P Rankin

**Affiliations:** 1 Memory and Aging Center, University of CaliforniaSan Francisco, California; 2 Department of Neurology, University of CaliforniaSan Francisco, California; 3 Department of Neurology, University HospitalBasel, Switzerland; 4 Memory Clinic, University Center for Medicine of Aging, Felix-Platter HospitalBasel, Switzerland; 5 Department of Radiology, University of CaliforniaSan Francisco, California; 6 Magnetic Resonance Imaging Unit, San Francisco Veterans Affairs HospitalSan Francisco, California; 7 Department of Speech, Language and Hearing SciencesTucson, Arizona

**Keywords:** Affective perspective taking, dementia, empathy, infero-lateral temporal cortex, neurodegeneration, semantic self-knowledge, unawareness, voxel-based morphometry

## Abstract

**Background:**

Neuroimaging studies examining neural substrates of impaired self-awareness in patients with neurodegenerative diseases have shown divergent results depending on the modality (cognitive, emotional, behavioral) of awareness. Evidence is accumulating to suggest that self-awareness arises from a combination of modality-specific and large-scale supramodal neural networks.

**Methods:**

We investigated the structural substrates of patients' tendency to overestimate or underestimate their own capacity to demonstrate empathic concern for others. Subjects' level of empathic concern was measured using the Interpersonal Reactivity Index, and subject-informant discrepancy scores were used to predict regional atrophy pattern, using voxel-based morphometry analysis. Of the 102 subjects, 83 were patients with neurodegenerative diseases such as behavioral variant frontotemporal dementia (bvFTD) or semantic variant primary progressive aphasia (svPPA); the other 19 were healthy older adults.

**Results:**

bvFTD and svPPA patients typically overestimated their level of empathic concern compared to controls, and overestimating one's empathic concern predicted damage to predominantly right-hemispheric anterior infero-lateral temporal regions, whereas underestimating one's empathic concern showed no neuroanatomical basis.

**Conclusions:**

These findings suggest that overestimation and underestimation of one's capacity for empathic concern cannot be interpreted as varying degrees of the same phenomenon, but may arise from different pathophysiological processes. Damage to anterior infero-lateral temporal regions has been associated with semantic self-knowledge, emotion processing, and social perspective taking; neuropsychological functions partly associated with empathic concern itself. These findings support the hypothesis that—at least in the socioemotional domain—neural substrates of self-awareness are partly modality-specific.

## Introduction

Impaired self-awareness, that is, an inaccurate subjective evaluation of one's trait or state relative to a more objective measurement, has been reported in various neuropsychiatric disorders, including neurodegenerative diseases (Orfei et al. 2008). It can involve the inadequate awareness of one's disease state (disease unawareness) or focally impaired self-reflective abilities in a specific modality, such as body sensation, various domains of cognition, or one's characteristic traits and attitudes (modality-specific unawareness) (Clare 2004b; Ecklund-Johnson and Torres 2005; David et al. 2012). These specific modes of self-awareness, and the objective evidence against which one's subjective self-evaluation is compared, are on a continuum from simple and concrete to highly abstract. Current evidence suggests that the cognitive processes required for different levels of self-awareness are likely represented in iterative stages in the brain, performed by subcortical and cortical networks (Schmitz and Johnson 2007; Craig 2009; Fleming and Dolan 2012).

In patients with neurodegenerative diseases such as Alzheimer's disease (AD) and behavioral variant frontotemporal dementia (bvFTD), impaired awareness is common, though it differs in modality and degree (Ecklund-Johnson and Torres 2005; Rankin et al. 2005; Hornberger et al. 2012). Many AD patients are highly aware of their cognitive deficits early in the disease, but all patients show increasingly inaccurate self-evaluation as the disease progresses (Ecklund-Johnson and Torres 2005). In contrast, early loss of self-awareness is a central feature of bvFTD (Neary et al. 1998). Typically, bvFTD patients describe their personality traits less accurately and are less aware of their specific behavior deficits than AD patients (Eslinger et al. 2005; Rankin et al. 2005; Salmon et al. 2008; Hornberger et al. 2012). bvFTD patients may also be less aware of their cognitive deficits than AD patients, even when they are less cognitively impaired (Williamson et al. 2010). In fact, bvFTD patients can display substantial deficits in self-awareness before showing measureable cognitive impairments (Lee et al. 2012), suggesting that self-awareness involves factors beyond the domains tested in a standard neuropsychological battery. This also suggests that the focal anatomy affected early in bvFTD may be more directly involved in self-awareness than the anatomy affected early in AD.

Results of functional and structural neuroimaging studies of self-awareness deficits in neurodegenerative disease generally confirm these hypotheses (Zamboni and Wilcock 2011). Results, however, are divergent across studies, likely due to methodological and conceptual differences such as the modality of self-awareness studied, the assessment methods used, or the sample's characteristics (Markova et al. 2005; Zamboni and Wilcock 2011). While some studies found correlations between self-awareness deficits and right frontal dysfunction (Starkstein et al. 1995; Mendez and Shapira 2005; McMurtray et al. 2006) and right ventro-medial atrophy (Rosen et al. 2010), others found correlations with lateral temporo-parietal (Salmon et al. 2006; Ruby et al. 2009) or anterior temporal dysfunction (Ruby et al. 2007), and right posterior temporal atrophy (Zamboni et al. 2010). These divergent results may indicate that self-awareness involves a large-scale supramodal neural network (Schmitz and Johnson 2007; Legrand and Ruby 2009), as reported in functional neuroimaging studies investigating the self in healthy individuals, that comprises the medial prefrontal cortex, precuneus/posterior cingulate gyrus, temporo-parietal junction, and temporal poles (Legrand and Ruby 2009).

Most previous neuroimaging studies of self-awareness in neurodegenerative disease have focused on whether patients were able to accurately estimate their level of cognitive functioning (Zamboni and Wilcock 2011). Studies examining patients' capacity to accurately evaluate their own personality traits are more rare (Zamboni and Wilcock 2011; Hornberger et al. 2012), and no studies have investigated the neural basis of patients' loss of self-awareness regarding a complex socioemotional characteristic such as their capacity to behave empathically toward others. Empathy is a well-characterized, complex social behavior, involving the subjective emotional feelings induced by others' emotions, the ability to differentiate between the feelings one experiences and the feelings expressed by others, and mental flexibility (Decety and Jackson 2004). Despite this complexity, healthy individuals are able to represent their own level of empathy fairly accurately, indicating that this information is normally accessible to awareness. Examining the neural substrates of self-awareness for this type of complex behavioral trait could provide information to better dissociate modality-specific from supramodal neural processes underlying self-awareness.

Previous neuroimaging studies have examined impaired self-awareness independent of its directionality, despite the fact that patients can show highly divergent patterns (Michon et al. 1994; Rankin et al. 2005; Tranel et al. 2010; Zamboni et al. 2010), with some patients overestimating their level of functioning (“polishers”) and others underestimating it (“tarnishers”). Rather than reflecting a continuum, being overcritical or under critical may reflect divergent pathophysiological processes, thus this should be investigated independently.

In this study, we asked whether either overestimation or underestimation of one's capacity for empathic concern predict specific patterns of focal brain damage in a large sample of neurodegenerative disease patients and healthy older adults. To answer this question, we separated the sample into “polisher” and “tarnisher” subsamples based on the subject-informant discrepancy method, using the Interpersonal Reactivity Index (IRI) (Davis 1983). Within each of these two subsamples, discrepancy measures were then correlated with structural MR images using voxel-based morphometry (VBM) across the whole brain. We also examined the degree to which the anatomy underlying self-awareness of empathic concern corresponds to the neural correlates of empathic concern itself and the neural correlates of affective perspective taking (Davis 1983), a cognitive capacity related to empathic concern (Davis 1983; Sollberger et al. 2012).

## Materials and Methods

### Subjects

We studied 102 subjects, including 83 patients diagnosed with one of five neurodegenerative diseases and 19 healthy normal controls. Of the 83 patients, 28 patients met the research diagnostic criteria for behavioral variant frontotemporal dementia (bvFTD) (Rascovsky et al. 2011), 16 met criteria for semantic variant primary progressive aphasia (svPPA) (Gorno-Tempini et al. 2011), 4 met criteria for nonfluent variant primary progressive aphasia (nfvPPA) (Gorno-Tempini et al. 2011), 23 met criteria for AD (McKhann et al. 1984), and 12 met criteria for corticobasal syndrome (CBS) (Boxer et al. 2006). Patients with a Clinical Dementia Rating (CDR) score of >2.0 (i.e., moderate dementia) were excluded because they were presumed to be unable to complete the IRI questionnaire describing themselves in a valid manner due to the severity of their cognitive deficits.

Nineteen older normal controls (NC) were recruited through advertisements in local newspapers and talks at local senior community centers. For inclusion, subjects had to have a normal neurologic exam, CDR = 0, Mini-Mental State Examination (MMSE) ≥28/30, and verbal and visuospatial delayed memory performance ≥ the 25th percentile.

There were several reasons for including patients from different diagnostic groups as well as NCs in the study. First, greater variance of both levels of self-awareness and gray matter volume increased the statistical power to detect brain–behavior relationships across the whole brain. Second, inclusion of NCs ensured that the normal end of the regression line was represented in all analyses, regardless of the brain region or behavior in question. Third, because socioemotional self-awareness might be mediated by several brain structures, inclusion of subjects with different brain atrophy patterns but similar levels of self-awareness maximized our ability to identify multiple parts of a potential neural network.

All subjects underwent neuropsychological testing with a comprehensive battery that has been described in detail elsewhere (Rosen et al. 2002).

All subjects were required to have an informant to corroborate their daily functioning. Informants were typically a relative who lived with the subject, and were required to have known the subject for more than 5 years. The subjects and their informants signed an institutional review-board-approved research consent form to participate in the study.

### Interpersonal Reactivity Index

The IRI is a questionnaire measure of empathy consisting of four 7-item subscales (empathic concern, perspective taking, fantasy, and personal distress) (Davis 1983). There is evidence that empathy as measured by the IRI is hierarchically organized, with one general dimension at the apex, primarily consisting of empathic concern, which is significantly related to different dimensions of social skills, and correlates nearly perfectly with the general latent construct of empathy (Cliffordson 2002). Accordingly, we considered the empathic concern subscale score as the best measure of participants' empathy. All subjects were asked to fill out the questionnaire describing their current level of empathic concern. Informants were asked to fill out the questionnaire twice, describing the subjects' current level of empathic concern as well as the subjects' level of empathic concern before the onset of disease. Informants describing NCs' past level of empathic concern were asked how the NCs' empathic concern was 5 years ago. In dementia research, collecting IRI data from caregivers and others who know the patient well is an effective and reliable method for assessing levels of empathy (Rankin et al. 2006). Questionnaires were completed within 3 months of magnetic resonance imaging (MRI) scan in the patient sample (median time span = same day; range, 0–69 days).

### Socioemotional self-awareness

To obtain an index of subjects' socioemotional self-awareness, we generated discrepancy scores by subtracting the informant's rating of the subject's current empathic concern (considered the most accurate rating) from the subject's self-rating of his/her current empathic concern. Thus, scores close to zero indicated that the subject's self-awareness was accurate, while scores farther from zero suggested greater inaccuracy. The directionality of the discrepancy score indicated whether subjects overestimated (positive value) or underestimated (negative value) their level of empathic concern relative to the informants' rating. To avoid spurious brain–behavior correlations due to extreme discrepancy scores, we converted discrepancy scores to *z*-scores based on subjects' mean and standard deviation (SD) and checked for *z*-scores above +3 SDs and below −3 SDs, though none were found and no subject was excluded on this basis.

### Change in empathic concern

Some subjects in the study were expected to have undergone significant changes (typically decreases) in their level of empathic concern in the past 5–10 years due to neurologic disease. Such changes may have directly influenced the accuracy of subjects' self-description of the trait (Clare 2004a). To account for this potentially confounding effect, change in empathic concern score was generated by subtracting the informant's rating of the subject's current empathic concern from the informant's rating of the subject's premorbid empathic concern. This score was included as a covariate in the VBM analysis removing potential confounds.

### Separation into polisher/neutral and tarnisher/neutral subsamples

The sample was separated into polisher/neutral and tarnisher/neutral subsamples based on value and directionality of the subjects' discrepancy scores. Subjects with discrepancy scores above the mean − ½ SD were part of the polisher/neutral sample (*n* = 69). Subjects with discrepancy scores below the mean + ½ SD were part of the tarnisher/neutral sample (*n* = 72). Consequently, subjects with discrepancy scores close to the mean (“neutrals”) were part of both groups. “Neutrals” were included in both groups to retain the naturally occurring variability in discrepancy scores and gray matter volumes in the statistical parametric mapping (SPM) group analyses, increasing power to detect neural substrates of over—and underestimation of one's empathic concern across the whole brain. Notably, all seven diagnostic groups included “neutrals,” suggesting high variability in gray matter volumes within the “neutrals.” Also, given that the identification of “polishing” and “tarnishing” is a clinical feature denoting an extreme behavior without a clear quantitative boundary, the conservative approach was to include the neutrals in both groups so that we did not artificially designate where normal self-awareness became “polishing” or “tarnishing.”

### Structural MRI

MRI scans were obtained on a 1.5-T Magnetom VISION system (Siemens Inc., Iselin, NJ) equipped with a standard quadrature head coil. A volumetric-magnetization-prepared, rapid-gradient echo MRI (MPRAGE, TR/TE/TI = 10/4/300 msec) was used to obtain T1-weighted images of the entire brain, 15° flip angle, coronal orientation perpendicular to the double-spin echo sequence, 1.0 × 1.0 mm^2^ in-plane resolution, and 1.5-mm slab thickness.

### Voxel-based morphometry

VBM preprocessing and analyses were performed using the SPM5 software package (Welcome Department of Cognitive Neurology, London; http://www.fil.ion.ucl.ac.uk/spm) running on MATLAB 7.1.0 (Math Works, Natick, MA). In all preprocessing steps, SPM5 default parameters were kept, except for the morphological filtering step, in which the light cleanup procedure was used. More anatomically precise intersubject registration was then performed with the Diffeomorphic Anatomical Registration through Exponentiated Lie algebra (DARTEL) toolbox (Ashburner 2007) by warping each subject's image to a template created from 50 additional older NC. Spatially normalized, segmented, and modulated gray matter images were smoothed with a 12-mm FWHM isotropic Gaussian kernel.

### VBM analyses of socioemotional self-awareness

Covariates-only (multiple regression design) statistical analyses were used to determine the relationship between discrepancy *z*-scores and smoothed gray matter volumes in the polisher/neutral sample (negative correlation) and in the tarnisher/neutral sample (positive correlation). Age, gender, MMSE (as a proxy for disease severity), and total intracranial volume (TIV) were entered as covariates into all designs. The resulting statistical parametric (SPM) map was thresholded at voxel-wise *P* < 0.001, and then corrected for multiple comparisons at *P* < 0.05 based on cluster extent and a custom-fit error distribution determined by 1000 permutations of the data (Wilson et al. 2010). Resulting SPM T-maps were superimposed on the Montreal Neurological Institute (MNI) single subject brain using automated anatomical labeling (AAL) included in the MRIcron software package (http://www.sph.sc.edu/comd/rorden/mricro.html). The following two statistical analyses were performed for identifying neural substrates of overestimation and underestimation of one's empathic concern:

#### Main effect analysis (voxel-wise regression of gray matter volume on empathic concern discrepancy score)

To identify neural correlates across all diagnostic groups, the empathic concern discrepancy score was correlated with smoothed gray matter volume, using a one-tailed t-contrast, adjusting for age, gender, MMSE, and TIV.

#### Analysis removing potential confounds (voxel-wise regression of gray matter volume on empathic concern discrepancy score controlling for *diagnostic group effects* and *amount of change*)

In order to perform an error-check control for potential co-atrophy effects, we parameterized each diagnosis (0 = *no*, 1 = *yes*) and entered all six diagnostic groups into the design matrix as confounding covariates (using five dummy variables to represent the six groups) (please see Rankin et al. 2009; Sollberger et al. 2009, and Data S1 for rationale and additional methodological details).

Change in empathic concern score was also included as a covariate to remove the effects of actual change from awareness of change.

We accepted a level of significance of *P* < 0.001 uncorrected for multiple comparisons within the brain areas of interest previously identified in the *Main effect analysis*, and *P* < 0.05 (corrected for family-wise error) for areas outside of these regions of interest.

Complementary to the univariate *Analysis removing potential confounds*, a multivariate error check was conducted to rule out the possibility of co-atrophy errors (please see Rankin et al. 2009; Sollberger et al. 2009, and Data S1 for rationale and additional methodological details).

To examine the degree to which self-awareness relies on the same neural regions as empathic concern or perspective taking in order to better characterize the processes involved, VBM analyses of the informant-based empathic concern score and affective perspective taking score (another IRI subscale designed to measure cognitive elements of empathy; Davis 1983) were additionally performed in the whole sample (*N* = 102). Both scores were positively correlated with smoothed gray matter volume, using a one-tailed t-contrast, adjusting for age, gender, MMSE, and TIV. Each of the two T-maps was separately overlaid on the T-map of self-awareness.

## Results

### Behavioral results

An omnibus analysis of variance using a general linear model with an alpha level of <0.05 showed significant differences in age and gender across diagnostic groups (Table 1). Significant differences in empathic concern scores—*F*(7, 94) = 5.44, *P* < 0.0001—and empathic concern discrepancy scores—*F*(7, 94) = 4.61, *P <* 0.001—were found across diagnostic groups. Post hoc pairwise comparisons based on a Dunnett-Hsu test showed that bvFTD and svPPA patients were on average both significantly less empathic and less aware of their level of empathic concern than NCs (*P* < 0.05). On average, these patients overestimated their level of empathic concern relative to informants' reports.

**Table 1:** Characteristics of subjects classified by diagnostic group.

Characteristics	bvFTD (*n* = 28)	svPPA (*n* = 16)	nfvPPA (*n* = 4)	AD (*n* = 23)	CBS (*n* = 12)	NC (*n* = 19)	*F*-value_(df)_
Age	62.4 (8.2)^2^	61.8 (6.7)^2^	62.0 (9.4)	63.3 (10.3)^2^	66.8 (9.2)	71.3 (7.5)	3.68^1^_(5, 96)_
Education	16.4 (3.0)	16.9 (3.6)	16.0 (0)	16.0 (3.1)	14.5 (2.0)	17.6 (3.1)	1.62_(5, 92)_
Gender (M/F)	21/7	10/6	2/2	15/8	4/8	7/12	*χ*^2^_(5, *N* = 96)_ = 10.53^1^
MMSE (0–30)	25.9 (4.7)	25.3 (5.5)	27.0 (3.6)	19.9 (6.3)^2^	22.6 (7.1)^2^	29.6 (0.7)	8.39^1^_(5, 96)_
CDR box score (0–18)	5.8 (2.9)^2^	4.0 (3.0)^2^	1.8 (2.4)	4.4 (2.3)^2^	4.3 (3.7)^2^	0.1 (0.2)	11.35^1^_(5, 95)_
NPI total (0–144)	38.4 (23.1)^2^	28.3 (19.7)^2^	5.5 (5.5)	12.9 (13.5)	22.9 (16.1)	0.0 (0)	5.25^1^_(7, 72)_
GDS (0–30)	8.0 (6.4)^2^	6.1 (3.0)	3.5 (2.4)	7.5 (4.8)^2^	11.7 (9.7)^2^	2.1 (2.2)	2.62^1^_(7, 73)_
IRI-EC score (0–56)	21.0 (6.6)^2^	20.2 (8.8)^2^	29.5 (4.4)	28.3 (5.1)	24.4 (6.4)	28.8 (3.0)	5.44^1^_(7, 94)_
IRI-EC discrepancy score	5.8 (7.1)^2^	7.7 (9.6)^2^	−2.0 (3.9)	−1.2 (5.1)	0.1 (5.5)	−0.8 (4.4)	4.61^1^_(7, 94)_

Positive empathic concern discrepancy scores indicate that the subjects overestimated their level of empathic concern, whereas negative scores indicate that the subjects underestimated their level of empathic concern. *F*-statistics are derived from general linear models with an alpha level of <0.05. bvFTD, behavioral variant frontotemporal dementia; svPPA, semantic variant primary progressive aphasia (PPA); nfvPPA, nonfluent variant PPA; AD, Alzheimer's disease; CBS, corticobasal syndrome; NC, normal controls; MMSE, Mini-Mental State Examination; CDR, Clinical Dementia Rating; NPI, Neuropsychiatric Inventory; GDS, Geriatric Depression Scale; IRI-EC, Interpersonal Reactivity Index Empathic Concern. Data are mean ± SD.

1 *P* < 0.05.

2 *P* < 0.05 versus NCs based on post hoc Dunnett-Hsu test.

#### Reliability of subjects' self-rating

Because many patients in this study were cognitively impaired, some might not have been able to provide a coherent, meaningful response to the self-report questionnaire. Previous studies that used self-ratings of neurodegenerative disease patients showed that patients' self-ratings of current functioning were on average close to their premorbid level of functioning (Rankin et al. 2005; Ruby et al. 2007), suggesting inaccurate, but not inconsistent patients' self-ratings. Accordingly, self-ratings of patients with high discrepancy scores (i.e., poor self-awareness) might still be understood as reliable (i.e., representing the patient's actual opinion, rather than random test error), if their ratings are close to informants' ratings of patients' premorbid empathic concern. Self-ratings of patients with either bvFTD or svPPA, the two patient groups showing the most impaired self-awareness, were close to their premorbid level of empathic concern according to informant report (*m* = −0.25 ± 6.1). These patients' self-ratings were as close to their premorbid level of empathic concern as the NCs` self-ratings were to their estimated level of empathic concern 5 years previously, *t*(61) = −0.04, *P* = 0.97, suggesting that bvFTD and svPPA patients rated their current level of empathic concern inaccurately, but in a valid manner.

### Neuroimaging results

#### Neural correlates of overestimation of one's empathic concern (polisher/neutral sample, *n* = 69)

**Figure 1:**
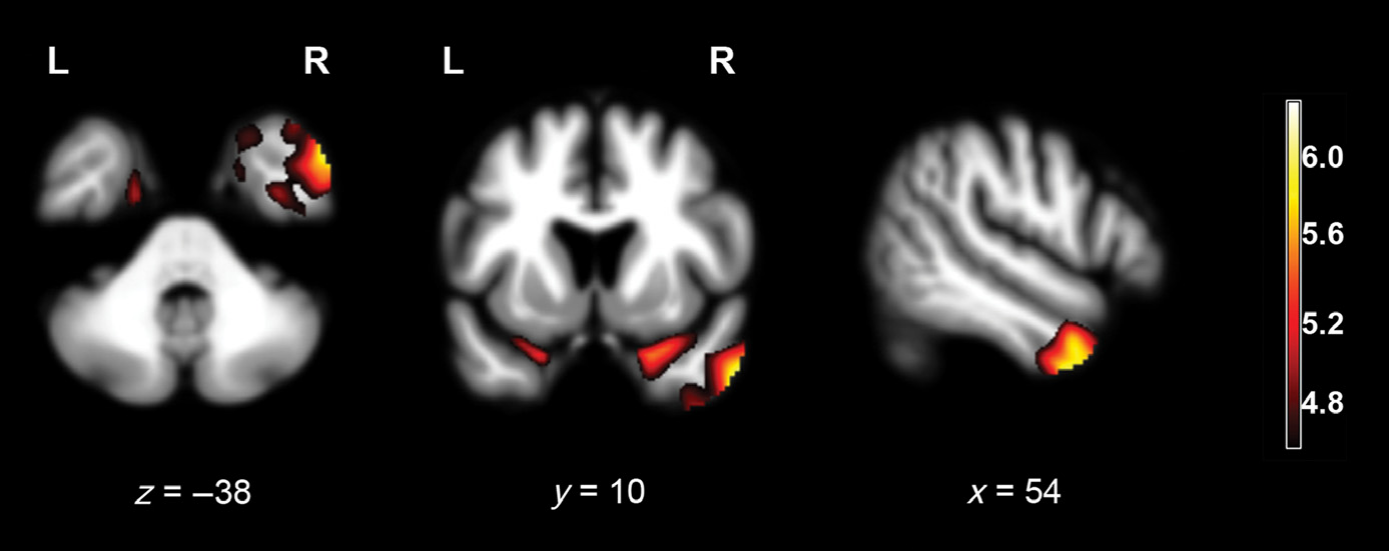
Results of the Main effect analysis of overestimation of one's empathic concern, superimposed on axial (*z* = −38), coronal (*y* = 10), and sagittal (*x* = 54) slices of a whole-brain template derived from normal controls. Red-yellow colored areas represent regions where atrophy negatively correlated with the discrepancy empathic concern score, adjusting for age, gender, Mini-Mental State Examination (MMSE), and total intracranial volume (TIV) (*P*_FWE_ < 0.05).

In the *Main effect analysis*, empathic concern discrepancy score correlated negatively with predominantly right-hemispheric gray matter volumes including the inferior and medial temporal gyri (close to the temporal pole), temporal poles, anterior fusiform gyrus, and anterior parahippocampus (*P*_FWE_ < 0.05; Table 2, Fig. 1). Please find the scatterplot of the most significant peak voxel's gray matter volumes at the right inferior temporal gyrus and empathic concern discrepancy score in the Data S1.

**Table 2:** Neural substrates of one's socioemotional overestimation (*n* = 69).

Anatomic region	mm^3^	*x*	*y*	*z*	*t*-value	*β*-weight
Main effects (critical threshold: 4.57)						
R inferior temporal gyrus	115,864	60	6	−34	6.24	−0.40*
R inferior temporal gyrus	″	46	4	−50	5.78	−0.27
R parahippocampal gyrus	″	24	14	−30	5.56	–
R superior temporal pole	″	36	30	−24	5.55	–
R superior temporal pole	″	30	20	−28	5.52	–
R superior temporal pole	″	28	20	−30	5.50	–
R cerebellum	″	28	−24	−28	5.32	–
R insula	″	42	−4	−10	5.10	–
R fusiform gyrus	″	40	−8	−34	5.01	–
R fusiform gyrus	″	34	−10	−50	5.01	0.27
R medial temporal pole	″	44	20	−40	5.00	–
R inferior orbital gyrus	″	30	21	−25	4.87	–
R parahippocampal gyrus	″	12	−2	−24	4.58	–
R parahippocampal gyrus	″	14	0	−24	4.58	–
L superior temporal pole	3152	−22	10	−28	5.19	–
L fusiform gyrus	″	−18	−8	−40	4.98	–
L fusiform gyrus	″	−30	−36	−14	4.64	−0.41*
Analysis removing potential confounds (critical threshold: 3.24)						
R inferior temporal gyrus	3648	54	4	−42	4.28	n/a

*Main effects*: regions where empathic concern discrepancy score negatively correlated with gray matter volumes, adjusting for age, gender, Mini-Mental State Examination (MMSE), and total intracranial volume (TIV) (corrected for family-wise error [FWE] across the whole brain at a significance level of *P* < 0.05). *Analysis removing potential confounds*: regions where empathic concern discrepancy score negatively correlated with gray matter volumes, adjusting for change in empathic concern score, diagnostic groups, age, gender, MMSE, and TIV (*P* < 0.001, uncorrected). Locations of clusters (mm^3^) are reported in the MNI reference space. – indicates region did not survive (*P* < 0.20) the modified backward selection procedure.

* *P* < 0.05.

Notably, there was some overlap in our superior temporal pole results with frontal insular regions in the right lateral orbitofrontal cortex (OFC). This finding, though, is probably spurious, because of the applied smoothing level and the fact that atrophy of both, the temporal poles and the lateral OFC, are common in patients with bvFTD (Seeley et al. 2008), rendering these regions highly susceptible for a “co-atrophy error.”

When diagnostic groups and change in empathic concern score were added as covariates to the design matrix (*Analysis removing potential confounds*), empathic concern discrepancy score correlated only with gray matter volumes of the right inferior temporal gyrus at a significance level of *P* < 0.001, uncorrected for multiple comparisons (Table 2).

Of note, empathic concern discrepancy score correlated strongly with change in empathic concern score (*r* = −0.68), supporting our approach to include change in empathic concern score as a covariate to remove the effects of actual change from awareness of change. Notably, there was a low level of collinearity in these variables, that is, variance inflation factors close to 2.

Finally, to determine the unique contribution of each brain region related to overestimation of one's empathic concern, we performed backward stepwise linear regression analyses of empathic concern discrepancy score on the voxel values at each peak coordinate from the *Main effect analysis* using SAS 9.1 (for more detail, please see the Data S1). Peaks representing the right anterior inferior temporal gyrus, the left anterior fusiform gyrus, and the right anterior parahippocampus remained in the final model (Table 2), explaining 47% (*R*^2^_adj_) of the total variance of the empathic concern discrepancy score. This was a large increase in explained variance—*F*(4, 60) = 13.70, *P* < 0.001, *R*^2^-change: 42%—compared to the covariates-only model (i.e., age, gender, MMSE, and TIV) (*R*^2^_adj_ = 5%).

#### Neural correlates of underestimation of one's empathic concern (tarnisher/neutral sample, *n* = 72)

The correlation between tarnishers' empathic concern discrepancy score and gray matter volumes did not survive multiple comparisons correction in any brain region (*P*_FWE_ < 0.05), though at an uncorrected level of *P* < 0.001, it correlated with volumes of the left anterior hippocampus—*t* = 3.51; *x* (−26), *y* (−14), *z* (−14).

#### Overlaps between neural correlates of overestimation of one's empathic concern and neural correlates of empathic concern

**Figure 2:**
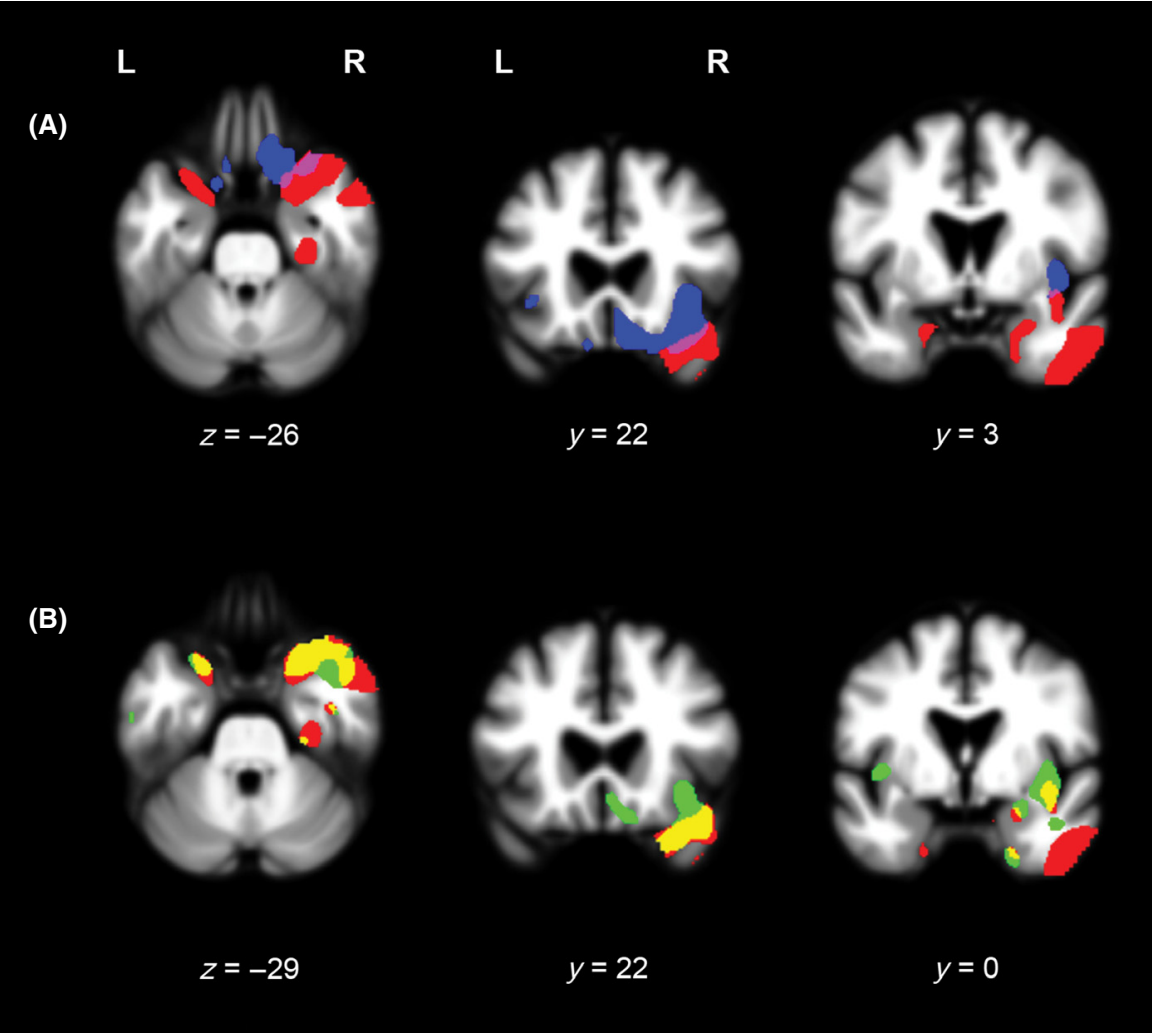
(A) Results of the Main effect analyses of overestimation of one's empathic concern (red) and empathic concern itself (blue), superimposed on axial (*z* = −26) and coronal (*y* = 22, *y* = 3) slices of a whole-brain template derived from normal controls. Red-colored areas represent regions where atrophy negatively correlated with the discrepancy empathic concern score, adjusting for age, gender, Mini-Mental State Examination (MMSE), and total intracranial volume (TIV) (*P*_FWE_ < 0.05). Blue-colored areas represent regions where atrophy positively correlated with the empathic concern score, adjusting for age, gender, MMSE, and TIV (*P*_FWE_ < 0.05). (B). Results of the Main effect analyses of overestimation of one's empathic concern (red) and affective perspective taking (green), superimposed on axial (*z* = −29) and coronal (*y* = 22, *y* = 0) slices of a whole-brain template derived from normal controls. Red-colored areas represent regions where atrophy negatively correlated with the discrepancy empathic concern score, adjusting for age, gender, MMSE, and TIV (*P*_FWE_ < 0.05). Green-colored areas represent regions where atrophy positively correlated with the affective perspective taking score, adjusting for age, gender, MMSE, and TIV (*P*_FWE_ < 0.05).

Both, overestimation of one's empathic concern and empathic concern itself correlated with gray matter volumes of parts of the right superior temporal pole and right posterior insula (*P*_FWE_ < 0.05; Table 3, Fig. 2). Additional areas along the right insular-orbitofrontal rim were involved in empathic concern but not self-awareness. Self-awareness involved additional right infero-lateral temporal regions and the left superior temporal pole compared to empathic concern itself (Table 2).

**Table 3:** Voxel-based morphometry analyses of empathic concern and affective perspective taking scores in the whole sample (*N* = 102).

Anatomic region	mm^3^	*x*	*y*	*z*	*t*-value
Empathic concern (critical threshold: 4.42)					
R mid-insula	105,864	42	6	0	6.01
R inferior orbital gyrus	″	36	19	−20	5.85
R anterior insula	″	37	20	−12	5.60
**R superior temporal pole**	″	**32**	**20**	−**24**	**5.35**
R superior orbital gyrus	″	13	18	−22	5.26
L inferior orbital gyrus	″	−14	13	−23	4.64
L superior orbital gyrus	″	−10	21	−24	4.59
L anterior insula	″	−37	21	−7	4.54
Affective perspective taking (critical threshold: 4.47)					
R inferior orbital gyrus	149,800	38	18	−20	6.33
R mid-insula	″	38	11	−5	6.16
**R superior temporal pole**	″	**38**	**19**	−**22**	**5.90**
**R superior temporal pole**	″	**30**	**19**	−**28**	**5.57**
**R inferior orbital gyrus**	″	**30**	**21**	−**25**	**4.94**
**R medial temporal pole**	″	**45**	**13**	−**29**	**4.85**
R amygdala	″	28	2	−17	4.83
R gyrus rectus	″	10	19	−16	4.81
**R parahippocampal gyrus**	″	**23**	**9**	−**27**	**4.75**
R inferior temporal gyrus	″	48	−16	−35	4.70
R superior orbital gyrus	″	11	18	−19	4.61
**R fusiform gyrus**	″	**27**	−**3**	−**40**	**4.53**
**L parahippocampal gyrus**	″	−**22**	**9**	−**25**	**5.19**
L mid-insula	″	−41	0	1	4.81
**L superior temporal pole**	″	−**32**	**15**	−**22**	**4.76**

Both empathic concern score and affective perspective taking score were positively correlated with gray matter volumes, adjusting for age, gender, MMSE, and TIV (*P*_FWE_ < 0.05). Overlaps of their neural substrates with the neural substrates of one's socioemotional overestimation are written in bold. Locations of clusters (mm^3^) are reported in the MNI reference space.

#### Overlaps between neural correlates of overestimation of one's empathic concern and neural correlates of affective perspective taking

Both, overestimation of one's empathic concern and affective perspective taking correlated with gray matter volumes of parts of the superior temporal poles, the right medial temporal pole, right anterior parahippocampal gyrus, frontal insular regions in the right inferior orbital gyrus, and right posterior insula (*P*_FWE_ < 0.05; Table 3, Fig. 2). Perspective taking itself also involved right medial OFC and frontal insula regions that were not also represented in the self-awareness map. Self-awareness involved right infero-lateral temporal regions that were not represented in the perspective-taking map (Table 2).

## Discussion

Our results suggest that while there are neural correlates of inaccurate socioemotional self-awareness in neurodegenerative disease patients, overestimation and underestimation of one's socioemotional capacity are not mediated by the same underlying structures. Although gray matter atrophy of predominantly right-hemispheric anterior infero-lateral temporal regions predicted overestimation of one's own capacity for empathic concern, no brain regions significantly predicted its underestimation. In addition, we found substantial overlaps between neural correlates of overestimation of one's empathic concern and empathic concern itself, providing a neuroanatomical basis for the clinical observation that the patients most lacking in empathy are commonly little aware of their poor empathy.

Overestimation of one's empathic concern (“polishing”) was predicted by predominantly right-hemispheric atrophy in anterior paralimbic and associative neocortical temporal brain regions and right posterior insula, with the most consistent and robust effects seen in the right anterior inferior temporal gyrus adjacent to the temporal pole, and the left anterior fusiform gyrus. Both brain regions have been associated functionally and structurally with amodal semantic knowledge (Binney et al. 2010). Retrieval of semantic knowledge, specifically semantic self-knowledge containing facts about one's personal characteristics, is likely critical for answering questions of the IRI Empathic Concern subscale (e.g., “I often have tender, concerned feelings for people less fortunate than me” or “I would describe myself as a pretty soft-hearted person”) (Davis 1983). Retrieval of episodic self-knowledge, however, a type of declarative memory primarily represented in the mesio-temporal and mesio-frontal brain regions, is not likely necessary to complete the IRI, as patients should not need to vividly re-experience past interpersonal events to complete the questionnaire (Burianova and Grady 2007).

In line with the neural substrates of overestimation of one's empathic concern, svPPA and bvFTD patients, the two diagnostic groups with atrophy patterns involving predominantly anterior temporal regions (Seeley et al. 2008; Brambati et al. 2009), significantly overestimated their capacity of empathic concern relative to healthy controls. These patients, especially in the case of predominantly right-hemispheric temporal atrophy, are known for behavioral disorders such as behavioral rigidity, obsessional behavior, disease unawareness, loss of empathy, as also for personality changes (Chan et al. 2009; Sollberger et al. 2009; Piguet et al. 2011). Yet lesion extension in these patients is much larger than the neural substrates of overestimation of one's empathic concern; in particular involving not only anterior temporal but—particularly in the case of bvFTD patients—preferentially ventromedial and insular structures (Seeley et al. 2008). Accordingly, we cannot tell if the brain regions depicted in this study are involved in the development of these patients' behavioral disorders.

### First-versus third-person perspective taking and self-awareness

Previous studies suggest that patients who are impaired in updating their socioemotional self-knowledge are more likely to rely on outdated premorbid self-knowledge (Rankin et al. 2005). Our findings support this hypothesis. Self-ratings of bvFTD and svPPA patients closely mirrored their premorbid levels of empathic concern as reported by an informant. Similar findings, reflecting impaired updating of one's socioemotional self-knowledge, were reported by Ruby et al. (2007) who asked bvFTD patients for their emotional reactions in social interactions.

Failure to update socioemotional self-knowledge, commonly associated with right-lateralized lesions of the anterior temporal lobes (Olson et al. 2013), may negatively affect one's socioemotional self-awareness (Conway 2005; Spreng and Mar 2012). Updating is partly based on feedback from the environment, which provides new information that can be used to adjust one's outdated self-knowledge. Thus, one's capacity to understand another person's thoughts and feelings likely underpins accurate socioemotional self-awareness, which is shaped in part by the opinion other people hold about one's own behavior in social settings (third-person perspective taking) (Ruby et al. 2007). The lack of susceptibility to external social inputs caused by impaired third-person perspective taking could prevent individuals from adjusting their first-person perspective. In this case, the underlying neural systems for knowing self and knowing other are likely to show substantial overlap. Support for a link between first-person and third-person perspective taking comes from functional neuroimaging studies in healthy subjects, showing vastly shared neural representations of self and other (Legrand and Ruby 2009). Moreover, there is also a link between third-person perspective taking and semantic knowledge. Third-person perspective taking draws upon one's semantic knowledge of the other persons' characteristics and one's self (Spreng and Mar 2012). Accordingly, the temporal poles, which have been associated with semantic knowledge (Binney et al. 2010), are part of the cerebral network commonly recruited in perspective-taking tasks in healthy individuals (Legrand and Ruby 2009). In this study, atrophy in the right > left temporal pole predicted overestimation of one's empathic concern. Moreover, these brain regions partially overlapped with the neural correlates of affective perspective taking, supporting the hypothesis that similar systems underpin one's capacity to take perspective on another person and on oneself (Ruby et al. 2007), and consequently mediate the accuracy of one's self-awareness.

Notably, our perspective taking scores are questionnaire-based measures of emotional perspective taking (Davis 1983), not to be equated with laboratory theory of mind tasks, primarily requiring inferential processing and/or attribution of agency to others (Carrington and Bailey 2009; Legrand and Ruby 2009). This might be why, the affective perspective taking score mapped only slightly onto some of the “classic” theory of mind regions such as the right ventromedial prefrontal cortex (Carrington and Bailey 2009; Legrand and Ruby 2009). Instead, it primarily mapped onto right anterior mesio-temporal regions associated with semantic appraisal and evaluation (Moll et al. 2005), which have also been found activated in theory of mind tasks (Carrington and Bailey 2009; Legrand and Ruby 2009; Mar 2011), and onto frontal insular regions in the right lateral orbitofrontal cortex associated with the “emotional salience network” (Seeley et al. 2007).

### Self-awareness in neurodegenerative disease patients

Results of previous functional and structural neuroimaging studies of impaired self-awareness in neurodegenerative disease are divergent (Zamboni and Wilcock 2011). Aside from the common problem of estimating association from small sample sizes, this divergence is likely due to differences in methodologies, including types of measures used to assess awareness (i.e., clinician rating of awareness, patient-informant discrepancy, judgment-performance discrepancy), diagnostic groups studied (restricting investigation to disease-affected brain regions), modalities of awareness examined (memory, personality traits, executive functions, activities of daily living), and statistical approaches (group comparisons versus correlational analyses either using region of interest or whole brain approach, adjusting for varying factors) (Clare 2004b; Markova et al. 2005; Zamboni and Wilcock 2011). Considering these caveats, our results were quite consistent with previous whole-brain VBM studies that used the patient-informant discrepancy method for measuring self-awareness of socioemotional behavior (Ruby et al. 2007; Zamboni et al. 2010; Hornberger et al. 2012), though these studies did not separate patients into polishers and tarnishers as our study did. In a bvFTD sample, Ruby and colleagues found that impaired self-awareness of emotions that were elicited in social settings related predominantly to left-sided hypometabolism of the superior temporal poles (Ruby et al. 2007). Because no atrophy correction was performed, this hypometabolism most likely co-occurred with atrophy in these regions. In a sample of FTD and CBS patients, impaired self-awareness for behavior as measured by the Frontal Systems Behavior Scale (Grace and Malloy 2001) related to atrophic right posterior temporal gray matter regions, including the inferior temporal gyrus and superior temporal sulcus (Zamboni et al. 2010). More recently, in a sample of FTD and AD patients, impaired self-awareness of behavior in social settings related to atrophic inferior temporal gyri (more posteriorly located than our peak regions) as well as to the left ventromedial prefrontal cortex (Hornberger et al. 2012).

In contrast, a structural VBM study in neurodegenerative disease patients using the judgment-performance discrepancy method found greater overestimation of cognitive performance related to atrophic right ventromedial prefrontal cortices (Rosen et al. 2010). These medial areas did not correlate with overestimation of empathic abilities in the present study, probably because cognitive capacities required for judging one's own cognitive performance such as inductive reasoning-having found related to these brain regions (Legrand and Ruby 2009; Fleming and Dolan 2012)-are not critical for estimating one's socioemotional behavior. However, there was some overlap in our superior temporal pole results with frontal insular regions in the right lateral orbitofrontal cortex. The inclusion of this brain region might be explained by its association with the “emotional salience network” (Seeley et al. 2007). This intrinsic network is critically involved in interoceptive-autonomic processing (Seeley et al. 2007), and may link emotional states and emotional awareness (e.g., Craig 2009).

### Modality-specific components of self-awareness

The discrepant results between studies of socioemotional and cognitive self-awareness support the hypothesis that the object of self-awareness likely influences the nature of self-related processing (Markova et al. 2005) and consequently its neural substrates (Zamboni and Wilcock 2011). Our data suggest that at least in the socioemotional domain, self-awareness may involve a modality-specific component in which the cognitive capacity itself, and the awareness of that cognitive capacity, engage the same neural system. In this study, neural substrates of empathic concern partially overlapped with neural substrates of overestimation of one's empathic concern, particularly in the right anterior superior temporal regions; brain areas also associated with empathy and processing higher level emotional and social information (Ruby and Decety 2004; Leiberg and Anders 2006; Olsson and Ochsner 2008; Olson et al. 2013). Empathic concern itself involves some of the cognitive capacities likely required for self-awareness, such as self-other distinction and perspective-taking capacities (Davis 1983; Decety and Jackson 2004; Leiberg and Anders 2006). Additional support for modality-specific neural substrates of self-awareness comes from a recent VBM study in neurodegenerative disease patients (Hornberger et al. 2012). In this study, neural substrates differed depending on the modality (e.g., motivation, emotion, social behavior) self-awareness related to. Similar to our findings, parts of these modality-specific neural substrates were close to brain regions associated with the respective modality (e.g., the amygdala was part of the neural substrates of self-awareness of one's emotion, but not part of neural substrates of self-awareness of one's social behavior) (Hornberger et al. 2012). To further explore potential modality-specific neural substrates of self-awareness, future studies should not only examine the neural basis of the respective self-awareness measure but also the neural basis of the modality to which it relates.

### Hemispheric lateralization of self-awareness

Similar to the majority of neuroimaging studies examining neural substrates of impaired self-awareness in various types of brain pathologies, such as neurodegeneration, stroke, schizophrenia, or traumatic brain injury (Orfei et al. 2008; Zamboni and Wilcock 2011), we found right lateralization of the neural substrates of overestimation of one's empathic concern. The variable lateralization patterns across studies might be partially due to the diversity of modalities of self-awareness studied, and also to the type of measures applied. For example, verbally demanding questions, likely engaging left hemispheric brain regions more than right-hemispheric brain regions (Knecht et al. 2000), might critically influence the lateralization of the neural substrates of the respective self-awareness measure. For instance, one's socioemotional self-awareness as measured by semantically demanding questions has previously been related to predominantly left-sided temporal pole activation in healthy controls (Ruby and Decety 2004).

Notably, in this study neural substrates of empathic concern itself were right-lateralized, whereas substrates of one's overestimation of empathic concern were found bilaterally with right-hemispheric predominance. Interestingly, bilateral involvement has been found in most neuroimaging studies of impaired self-awareness (Orfei et al. 2008; Zamboni and Wilcock 2011), pointing to a potentially critical link between self-awareness and parallel processing in bilaterally connected neural circuits.

### “Tarnishing” may be multifactorial

No brain region significantly predicted underestimation of one's empathic concern, which supported our hypothesis. As pointed out by others (Tranel et al. 2010), our data suggest that relationships between measures of self-awareness and other measures such as brain atrophy can be obscured by examining self-awareness measures independent of their directionality. Awareness of this issue is critical not only for interpreting previous neuroimaging studies of impaired self-awareness in neurodegenerative disease in which patients were not separated into those who polish (overestimate) and tarnish (underestimate) their functioning but also for designing and analyzing future studies.

One potential reason we did not find a structural brain basis for underestimation of one's empathic concern is reflected by the fact that tarnishers showed little change in their empathic concern relative to their premorbid level, likely resulting in restricted range of brain-behavioral relationships. Most likely, psychological factors such as subjects' and informants' personality and motivation, as well as social and contextual factors, influenced the informant-subject discrepancy measure (Clare et al. 2012). If tarnishing was part of a patient's general self-deprecating style, it could be related to psychiatric issues such as anxiety or depression, or a result of learned social behavior consistent with cultural factors. While tarnishing might still have a brain basis, this might be found in neurotransmitter activity or functional connectivity patterns, or so multifactorial that they could not be isolated to frank structural atrophy. It is also possible that brain regions underlying underestimation of one's empathic concern are widely distributed, not allowing strong correlations between single brain regions and measures of self-awareness.

### Limitations

Some of the primary caveats to the interpretation of our data are inherent in the VBM-technique and the whole-brain approach. First, because the VBM method is essentially based on an atrophy model that relies on the use of a clinically defined sample of subjects with diverse atrophy patterns, the extent to which results can be generalized beyond a study's population of interest is an issue of debate. However, this method has been used to accurately localize cognitive functions to brain areas in patients that had previously been identified in healthy controls using other, nonatrophy-based techniques (Amici et al. 2007), suggesting that generalization is often both possible and appropriate, in particular when working with large sample sizes as in this study. Nevertheless, the influence of disease-specific patterns of co-atrophy remains a potential confound. We addressed this issue by performing two additional analyses designed as error checks, which increased the likelihood that our results are not restricted to our study sample but are generalizable to normal brain function. Yet, it remains unclear how much normal aging and neural plasticity in the context of disease may limit such generalization. Second, the degree to which structural VBM is truly a whole-brain analysis is limited by the particular composition of the subject sample. This study intentionally included a large sample of patients with a diverse selection of diseases known collectively to affect most cortical structures in order to maximize sample-wide variability in both brain atrophy and behavior. Though our SPM ResMS maps suggested good variability throughout the cortex in our sample, it remains possible that some brain regions might have suffered from restriction of range and a corresponding loss of power to detect brain-behavior relationships, particularly in cases where only small numbers of subjects had atrophy to an important region.

Finally, our discussion of the clinical and neuroimaging results in the polisher and tarnisher samples are limited by the fact that the clinical phenomena appear in different patients, thus they cannot be directly compared within the same set of subjects.

## Conclusions

These data demonstrate that in neurodegenerative disease patients, overestimation (“polishing”) of one's socioemotional behavior, specifically the tendency to behave with empathic concern, is related to atrophy in predominantly right-hemispheric anterior infero-lateral temporal regions, thereby highlighting the critical role of semantic self-knowledge and perspective taking capacity for one's socioemotional self-awareness. In addition, we found a close association between socioemotional overestimation and socioemotional concern, implying that—at least in the socioemotional domain—neural substrates of self-awareness are partly modality-specific. Finally, we showed that one's socioemotional overestimation and underestimation are likely based on different pathophysiological constructs, implying that future studies should examine impaired self-awareness with careful attention to the direction of error.

## Supporting Information

Data S1 Supplementary Material.

Figure S1 Scatterplot of the Main effects' peak voxel's gray matter (GM) volumes at the right inferior temporal gyrus (60/6/–34) and empathic concern discrepancy score, adjusting for age, gender, MMSE, and TIV, using STATA 9.2.
