# Supplementary material for: Neural substrates of socioemotional self-awareness in neurodegenerative disease
Source: Brain Behav. 2014 Jan 13;4(2):201–14. doi: 10.1002/brb3.211 (PMC3967536; doi:10.1002/brb3.211)
Supplement: Data S1 — Supplementary Material. [file brb30004-0201-sd1.docx]

**SUPPLEMENTARY MATERIAL:**

*Analysis Removing Potential Confounds (voxel-wise regression of grey matter volume on Empathic Concern Discrepancy score controlling for diagnostic group effects and amount of change):*

Because the Main Effect Analysis is based on disease-related atrophy, it is possible to obtain statistical significance even if they only occur in one diagnostic group and do not represent a generalizable brain-behavior relationship (i.e., “coatrophy error”). Logically it is possible for this kind of illusory correlation to occur in any VBM analysis done on patients from multiple neurodegenerative disease groups, because if disease group membership predicts a region of atrophy (G→A) and also predicts poor performance on the behavior task (G→B), then that region of atrophy may appear to directly correlate with the behavior (A↔B), when that correlation is actually spurious (A←/→B). The results of this analysis show regions of atrophy significantly related to the discrepancy score only if they appear in more than one diagnostic group. However, these results must be considered in light of the main effects results because it will fail to identify any brain region that is legitimately related to the discrepancy score but is atrophied only in a single diagnostic group.

*Co-Atrophy Check (regression of Empathic Concern Discrepancy score on significant peak voxels’ grey matter volumes of the Main Effect Analysis):*

While the Analysis Removing Potential Confounds is univariate, not accounting for covariance across brain regions, the Co-Atrophy Check performs a direct, multivariate comparison of brain regions *to each other* in order to weed out spurious, multicollinear regions. Thus, this error check complements the Analysis Removing Potential Confounds. By performing backward stepwise linear regression analyses of Empathic Concern Discrepancy score on the voxel values at each peak coordinate from the Main Effect Analysis, we aimed to determine the unique contribution of each brain region related to one’s socioemotional self-awareness. Voxel probabilities of each subject at each peak voxel were extracted from the smoothed grey matter images, then analyzed together to determine their ability to independently predict discrepancy score in linear regression analyses, including age, gender, MMSE, and TIV as additional confounding predictors. We used the Allen-Cady modified backward selection technique ([Vittinghoff, et al., 2004](#_ENREF_1)), setting a very permissive inclusion threshold at *p*<.20 to ensure that brain regions showing at least a modest independent relationship to the discrepancy score remained in the model.

**References:**

Vittinghoff, E., Glidden, D.V., Shiboski, S.C., E., M.C. (2004) Regression Methods in Biostatistics: Linear, Logistic, Survival, and Repeated Measures Models. New York. Springer. 339 p.

**Figure legend**

Scatterplot of the Main Effects’ peak voxel’s grey matter (GM) volumes at the right inferior temporal gyrus (60/6/-34) and Empathic Concern Discrepancy z-scores, adjusting for age, gender, MMSE and TIV, using STATA 9.2.
