# Supplementary figures and images for: Neural substrates of socioemotional self-awareness in neurodegenerative disease
Source: Brain Behav. 2014 Jan 13;4(2):201–14. doi: 10.1002/brb3.211 (PMC3967536; doi:10.1002/brb3.211)

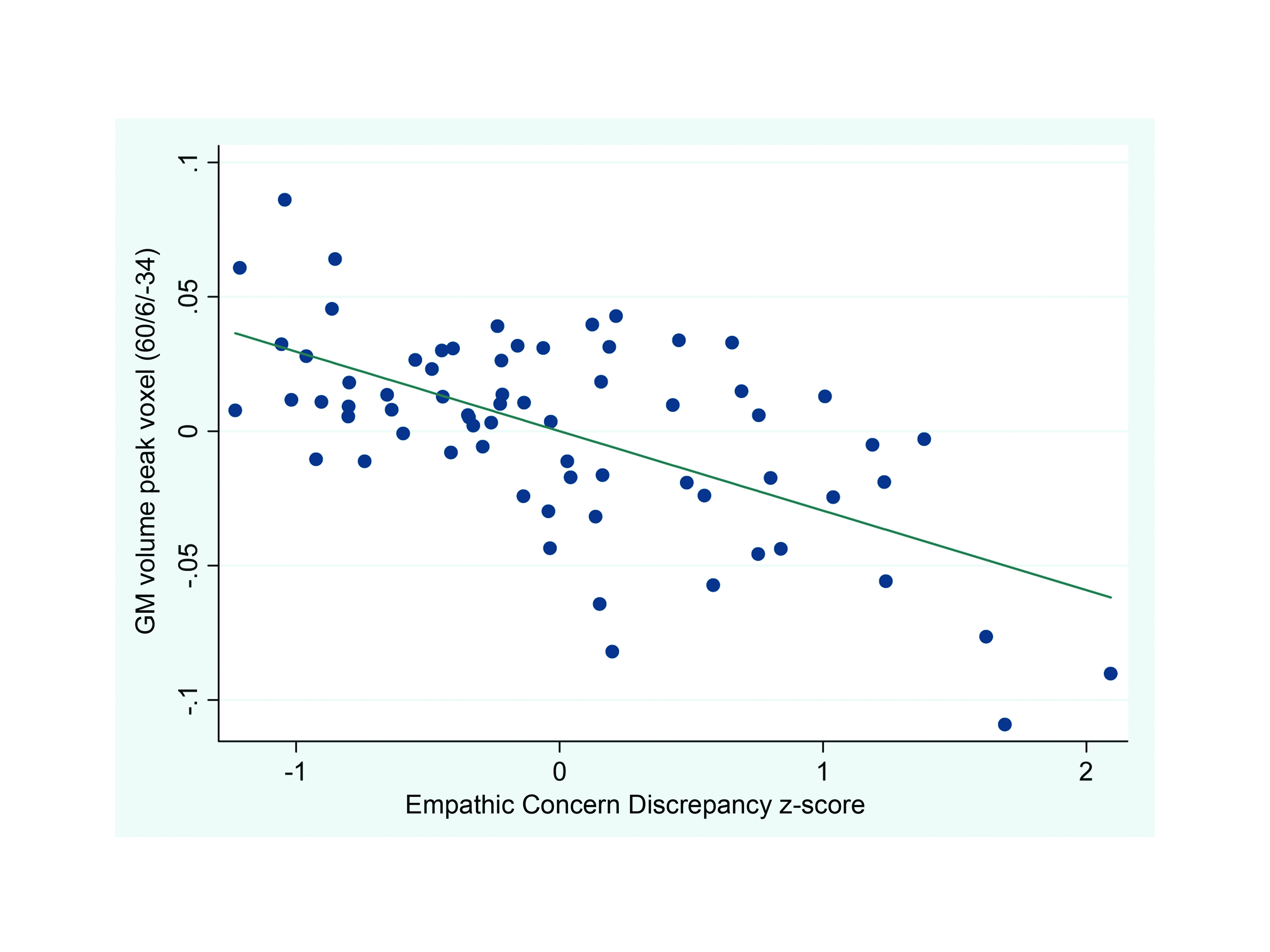

Supplement: Figure S1 — Scatterplot of the Main effects' peak voxel's gray matter (GM) volumes at the right inferior temporal gyrus (60/6/–34) and empathic concern discrepancy score, adjusting for age, gender, MMSE, and TIV, using STATA 9.2. [file brb30004-0201-sd2.tif]
